# Plasma paraoxonase-1 activity levels in patients with type 2 diabetes mellitus in Lagos State University Teaching Hospital, Lagos, Southwest Nigeria: a cross-sectional study

**DOI:** 10.11604/pamj.2023.45.40.36301

**Published:** 2023-05-17

**Authors:** Adeyemi Oluwaseun Dada, Uduak Akpan Ikpegbu, Lanrewaju Oluwaseun Okunowo, Adeola Olubunmi Ajibare, Mayowa Emmanuel Adekiitan, Halimat Oyindamola Shasore

**Affiliations:** 1 Department of Chemical Pathology, Lagos State University College of Medicine, Lagos, Nigeria,; 2 Department of Chemical Pathology, General Hospital, Lagos Island, Lagos, Nigeria,; 3 Department of Medicine, Cardiology Unit, Lagos State University Teaching Hospital, Lagos, Nigeria,; 4 Department of Chemical Pathology, Lagos State University Teaching Hospital, Lagos, Nigeria

**Keywords:** Paroxonase, diabetes mellitus, cardiovascular disease

## Abstract

**Introduction:**

paraoxonase 1 (PON1) is a high-density lipoprotein (HDL) associated enzyme that has anti-inflammatory, anti-atherogenic, and antioxidant functions. PON1 is noted to be a determinant of resistance to the development of atherosclerosis through hydrolysis of phospholipid and cholesteryl ester hydroperoxides. This study was designed to assess PON1 activity levels among patients with type 2 diabetes mellitus (T2DM) in Southwest Nigeria.

**Methods:**

this was a cross-sectional study done over a period of six months. A total of 138 participants; 69 with T2DM and 69 apparently healthy controls were recruited for this study. Fasting plasma glucose (FPG), HDL cholesterol (HDL-c), and PON1 activity were analyzed in the participants. The comparison of the mean between the groups of participants was assessed using the independent student t-test while the Mann-Whitney U test was used to compare two medians. The p-value was set at 0.05.

**Results:**

mean age for participants with T2DM was 54.90 ± 8.1 years and the healthy control group was 54.12 ± 8.4 years, with a p-value of 0.549. The male-to-female ratio was 0.47 for both participants with T2DM and healthy controls. Participants with T2DM had significantly higher median glucose concentration of 109.18 mg/dl compared with 82.58 mg/dl among controls, p-value <0.001. Median serum HDL-c was lower in diabetics compared to controls (52.66 mg/dl vs 57.92 mg/dl; p-value < 0.001). PON1 activity was lower in T2DM compared with that of the controls (690.11 pmol/min/ml vs 3379.7 pmol/min/ml; p-value <0.001). Paroxonase 1 showed a non-significant positive correlation with HDL-c and a negative correlation with FPG, and body mass index (BMI).

**Conclusion:**

these findings suggest that PON1 activity is lower in T2DM compared to healthy controls and a lower PON1 activity level was seen among female diabetics compared with the male diabetics.

## Introduction

Worldwide, according to an International Diabetes Federation regional estimate, 14% of the 384 million individuals living with diabetes mellitus are found in Africa [1]. Patients with type 2 diabetes mellitus (T2DM) have been reported to have increased risk of cardiovascular diseases (CVD) [2].

Dyslipidemia in patients with T2DM includes hypertriglyceridemia and subnormal high-density lipoprotein cholesterol (HDL-c) which contributes adversely to coronary heart disease (CHD) [3,4]. Low plasma HDL-c is one of the common lipid disorders among patients with T2DM in West Africa [5]. Furthermore, the anti-inflammatory, antioxidant, antithrombotic, and endothelial function-promoting activities of HDL have been studied [6,7].

Diabetes mellitus is characterized by hyperglycemia with Insulin resistance (IR) as one of the major underlying pathogenetic mechanisms [8]. This inadequate response by insulin-sensitive tissues to normal circulating levels of insulin leads to impairment in insulin-mediated suppression of hepatic glucose production, skeletal muscle glucose disposal, and inhibition of lipolysis. The consequence is relative hyperglycemia, increased plasma levels of non-esterified fatty acids (NEFAs) [9], oxidative stress, leading to impaired blood vessel functions, defective vascular reactivity, impaired regulation of thrombosis, and increased risk for vascular inflammation [10-12].

Insulin resistance impairs hepatic glucose production [13] and increased free fatty acid (FFA) delivery to the liver which increases hepatic very low density lipoprotein (VLDL) and triglyceride (TG) production [14]. Alterations in FFA metabolism and excessive release of FFA has been labelled as a major factor involved in the pathogenesis of hyperglycemia and Dyslipidemia [15]. Increased plasma TG leads to increased HDL clearance and decreased plasma HDL concentration [16-18]. High density lipoprotein molecule which can become oxidized [19] has lipid components which include paroxonase 1 (PON1) [20].

Impaired serum PON1 activity is associated with higher triglyceride and lower HDL-c concentration pointing to a likely mechanism connecting the enzyme with an increased risk of CVD [21]. Paroxonase 1 is a calcium-dependent enzyme [22] and circulates in the plasma exclusively in association with HDL [23], and possesses antioxidant properties [4,24]. Hyperglycemia leads to glycation of HDL-c and PON1 protein, thereby reducing its HDL binding and activity [22,25], with resultant increased cardiovascular complications, morbidity and mortality related to T2DM [26].

Among the proteins present on HDL-c, PON1 is positively correlated and the most effective protein [27-29]. Information on PON1 activity in individuals with T2DM is scanty in this locality hence the need to study levels of PON1 activity amongst them. This study aimed to determine the levels of PON1 activity among participants with T2DM and compare same with healthy adults in Southwest Nigeria.

## Methods

**Study design and setting:** this is a descriptive cross-sectional study conducted over a period of six months from July to December 2017. The participants with DM for this study were recruited from the Diabetes, Family Medicine, and Metabolic Clinics of Lagos State University Teaching Hospital (LASUTH), a state-owned urban tertiary hospital in Nigeria. The healthy control participants were recruited from members of staff and other members within the community. This hospital attends to a mixture of both the high, middle and low-income patients due to the affordability of services, therefore, making it a centre of choice for sampling. Lagos State is a cosmopolitan and mega-city in Southwest Nigeria, where virtually every tribe in Nigeria is well represented. In this city, there is a mixture of lifestyle patterns. These lifestyle patterns range from reduced activity levels due to the jobs many people engage in as well as those who engage in more active jobs like artisans. Transportation systems include roads, waterways, and rail. Other important lifestyle patterns include higher levels of indulgence in excessive consumption of alcoholic beverages, smoking habits, and unhealthy dietary preferences which are common in any megacity. These lifestyle changes encourage an increase in obesity and T2DM.

**Study population:** a total of 138 participants consisting of 69 type 2 DM and 69 apparently healthy age-matched controls were recruited for this study. This was determined using the formula: [30].


$$n=\frac{z^{2}p\left(1-p\right)}{d^{2}}$$

Where n = sample size, Z = Z statistic for a level of confidence at 95% (standard value of 1.96), P = expected prevalence or proportion (in proportion of one; a prevalence of 4.6% for T2DM based on Southwest studies) [31], d = precision/margin of error (precision of 5%). Inputting variables:


$$n=\frac{1.96^{2}*0.046\left(1-0.046\right)}{0.05^{2}}=67$$

In order to increase the validity of the study, an extra five percent (5%) was included to cater for dropouts to give a sample size of 69. An equal number (69) of apparently healthy subjects were recruited as controls. Participants included in the study were those diagnosed to have type 2 diabetes mellitus according to the defined criteria of a fasting plasma glucose level of 126 mg/dL (6.99 mmol/L) or greater, an HbA_1c_ level of 6.5% or greater, or a 2-hour post glucose load level of 200 mg/dL (11.1 mmol/L) or greater [32]. The patients gave consent to be recruited for the study and healthy participants who gave their consent were used as controls. Patients on insulin therapy, type 1 DM; patients with liver disease, renal failure, patients on drugs that can affect lipid metabolism e.g. thiazide, β-blocker, oestrogen, glucocorticoids, highly active antiretroviral therapy (HAART); patients with T2DM who are still actively engaged in smoking and excessive consumption of alcohol and those who did not consent were exempted from the study.

**Data collection:** participants were requested to respond to a self-administered questionnaire concerning information on biodata, presence/absence of diabetes, duration of diabetes, family history of diabetes, treatment details, complications, and other co-morbidities and previous laboratory results being used for follow-up. Anthropometric measurements (weight, height, abdominal circumference) were taken using calibrated weighing scales, standardized vertical tape and stretch elastic tape. Subjects were also required to have fasted overnight (10-12 hours) before coming to the clinic for venous blood sample collection. Venous blood was collected from the antecubital fossa using multiple sampling needles into vacutainers under aseptic conditions. Five milliliters (5 mls) of blood was collected into lithium heparin vacutainers for PON1 and lipid profile estimation, and three (3 mls) of blood were collected in a fluoride oxalate bottle for fasting plasma glucose estimation. The needles were discarded into biosafety boxes and other biohazard wastes were disposed of through the hospital medical waste disposal unit.

Analysis of fasting plasma glucose, plasma high-density lipoprotein cholesterol (HDL-C) and PON1 were done on the centrifuged and separated plasma obtained from the venous blood obtained from the participants. The activity of PON1 was analysed using a paraoxonase 1 activity assay kit using fluorometric method, with reagent kits from Bio Vision USA. Lipid estimation was done using quantitative-enzymatic-spectrophotometric method and was assayed using reagent kits from Biolabo SA, Maizy, France. Haemolysed, icteric or lipaemic samples were excluded from the analysis. Repeated thawing and freezing was avoided. Analysis was done in the Research Centre of the Lagos State University College of Medicine (MRC, LASUTH).

**Definitions:** body mass index was defined as weight divided by the square of the height.

**Statistical analysis:** data analysis was carried out using statistical package for social sciences version 26 (SPSS Inco, Chicago, IL). Normally distributed continuous variables were reported as mean and standard deviation (SD) while the continuous variables with non-parametric distribution were reported as median (interquartile range). Comparison of mean between the groups of participants was assessed using independent student t-test while Mann-Whitney U test was used to compare two medians. The p-value was set at 0.05.

**Ethical consideration:** ethical approval was obtained from the Research and Ethics Committee of the Hospital with approval number LREC/10/06/656. Written informed consent was obtained from all participants selected for the study after the study was explained to each participant in a language that they understand.

## Results

**General characteristics of the participants:** a total of 138 participants were recruited for the study. These comprised of 69 type 2 diabetes mellitus participants and 69 healthy controls. Mean age for the T2DM was 54.90 ± 8.1 years while the healthy control group had a mean age of 54.12 ± 8.4 years without any significant difference between the mean ages of the two groups with p-value of 0.549. The participants were sex-matched as indicated in Table 1. The male-female ratio was 0.47 for both T2DM and healthy controls. Body mass index was slightly higher among T2DM (25.65 ± 4.5 kg/m^2^) than the control subjects (25.48 ± 4.5 kg/m^2^) with no significant difference. Waist-hip ratio was lower among controls, however, there was no significant difference (Table 1). There was however a significant difference in both systolic and diastolic blood pressures among the T2DM compared with the healthy controls with the participants with T2DM having higher values (Table 1).

**Table 1:** demographic status of the study participants

Parameters	Diabetes (n=69) mean ± SD	Control (n=69) mean ± SD	p-value
Age	54.90 ± 8.1	54.12 ± 8.4	0.549
**Gender**			
Male	22(31.9)	22(31.9)	
Female	47(68.1)	47(68.1)	1.000
BMI	25.65 ± 4.5	25.48 ± 4.9	0.830
WH ratio	0.90 ± 0.1	0.89 ± 0.1	0.305
Systolic	128.20 ± 18.5	119.65 ± 10.0	0.001*
Diastolic	80.23 ± 10.3	76.38 ± 5.0	0.006*

* Statistically significant; BMI: body mass index; WH: waist hip

**Plasma glucose, HDL-c, and serum PON1 activity:** Table 2 shows the median comparison of plasma glucose, HDL-c, and serum PON1 activity among participants with T2DM and controls. Healthy controls had significantly higher HDL-c and PON1 activity than the T2DM counterparts. Median levels of glucose were higher among participants with T2DM.

**Table 2:** biochemical parameters: comparison of fasting plasma glucose, lipid profile and PON1 activity amongst the participants

Parameters	T2DM	Controls	p-value
	**n=69**	**n=69**	
	**Median (IOR)**	**Median (IOR)**	
FPG (mg/dL)	109.18 (79.8-165.2)	82.58 (77.3-92.3)	<0.001*
HDL-c(mgl/dL)	52.66 (45.2-59.5)	57.92 (50.2-65.6)	<0.023*
PON1(pmol/min/ml)	961.2 (690-1148)	3379.7(2772-3959)	<0.001*

* Statistically Significant; FPG: fasting plasma glucose; HDL-c: high density lipoprotein cholesterol; PON1: paroxonase 1

**PON1 activity in participants with controlled plasma glucose:** participants with T2DM who had controlled diabetes based on fasting plasma glucose of less than 130mg/dL had a higher median PON1 activity compared to the participants with uncontrolled glucose levels. Participants who had better control had PON1 activity of 973.87 (754-1178), while the participants with uncontrolled diabetes had a PON1 value of 918.07 (576-1063) with p-value of 0.189.

**PON1 activity among male and female participants with T2DM:** median PON1 activity among T2DM males was 1034.51 (750-1224) and females were 945.60 (657-1070) p=0.207; while control males had a median PON1 value of 3061.51 (2700-3796) compared to the females with 3580.74 (3054-4105), p=0.076. PON-1 was noted to have a weak and non-significant positive correlation with HDL-c and, a negative correlation with FPG, BMI, blood pressure, and waist hip ratio (WHR).

## Discussion

This study set out to assess PON1 activity among participants with T2DM and healthy controls. Our study showed that serum paroxonase 1 activity was significantly lower among participants with diabetes compared to the controls. We also found a higher BMI and FPG among participants with T2DM with an inverse relationship with PON1 suggesting that participants with a poorer clinical state have poorer anti-inflammatory and antiatherogenic tendencies.

This relationship aligns with some of the studies earlier noted. The mean age of both control and participants with T2DM in our study were similar with a comparable male-to-female ratio. In addition, we found that the HDL-c levels in controls were higher compared with the participants with T2DM. There have been different findings regarding the status and levels of PON1 in patients with T2DM.

According to a study by Adiga, Banawalikar, and Menambath, PON1 levels were increased significantly in patients with T2DM compared to controls [33]. On another note, some studies reported that control subjects had a higher HDL-c and PON1 activity when compared to patients with T2DM who had complications. They also noted that HDL-c was significantly decreased among T2DM without complications compared to healthy controls [34-36]. According to a study done in India with forty patients with T2DM an equal number of controls, a higher significant PON1 activity was recorded among the controls [36]. The different findings have prompted some researchers to further confirm the findings of these studies.

Paroxonase 1 was reported to be higher among African Americans compared to Caucasians and the same study noted that females of African American descent have a higher PON1 activity compared to their male counterparts and the younger females had higher PON1 activity when compared with older females. Males on the other hand have an increasing PON1 activity with age [10].

According to the study looking at the impact of gender on PON1, it was reported that females had significantly higher PON1 arylesterase and lactonase activities compared with the male participants and that gender had a strong impact on the contribution of PON1 to cardiovascular disease risk [37]. The female control participants in our study had a higher median PON1 activity than the male counterparts whereas the female participants with T2DM had a lower median PON1 activity compared to their male counterparts, thereby supporting the findings by Trentini *et al*. [37]. It is not known if there are factors in female T2DM which impair PON1 activity, therefore further studies will be required in this regard.

The role of PON1 in hydrolysing proinflammatory oxidized lipids thereby neutralizing the atherogenic properties have been reported and in addition, noted to reduce the accumulation of lipid peroxidation products by decomposition of hydrogen peroxide and lipid peroxides [10].

Other functions of PON1 include the protection of HDL and LDL from oxidation, promotion of macrophage cholesterol efflux and hydrolyzes of homocysteine thiolactone. A number of factors have been reported to affect the levels of PON1 and these include medications like lipid-lowering medications (statins and fibrates), vasoprin, rosiglitazone, and other antidiabetic medications have been noted to increase PON1 activity between 5 and 23%. Other medications like vitamins C and E were also reported to have modest increases on baseline PON1 activity. Dietary unsaturated fat, cholesterol, and polymorphisms (PON1-L55M and PON1-T-108C) also cause elevation of PON1. Factors that decrease PON1 activity includes dietary saturated fat, proinflammatory cytokines, oxidative stress, and smoking [38,39]. Our study is a hospital-based one and the participants were on some medications like antidiabetics, vasoprin, and some antioxidants. To this end, the effect of these medications on PON1 in these participants could not be verified. However, since the T2DM participants had a lower PON1 activity level, despite being on medications that may increase PON1 activity, they still had a significantly lower value compared to control participants who are not on any medications. This further strengthens our findings. Looking at the serum PON1 activity among diabetics, we noted that diabetics with a better glucose control had a higher PON1 activity. This supports the report that poor glycemic control has a negative impact on PON1 activity even among T2DM thereby reducing the benefits of PON1 in T2DM with poorly controlled glycemic status.

Our study is one of the few which had been done on PON1 in this environment, but is limited in not been able to completely eradicate all the possible factors that affect PON1.

## Conclusion

Our study supports previous findings which reported that participants with T2DM have lower PON1 activity compared with healthy controls; female controls have a higher PON1 activity than their male counterpart. Levels of PON1 activity showed an inverse relationship with BMI and FPG in the participants.

### What is known about this topic

- *Paraoxonase 1 (PON1) has both anti-inflammatory and anti-atherogenic properties;*
- *Divergent reports of PON1 activity levels in type 2 diabetics compared with healthy controls*.

### What this study adds

- *Type 2 diabetics have a lower PON1 activity levels compared with controls;*
- *Female controls have a higher PON1 activity levels while female diabetics have a lower PON1 activity level*.

## References

[1] International Diabetes Federation (IDF) (2013). IDF Diabetes Atlas 6^th^ edition.

[2] Ginsberg HN, MacCallum PR (2009). The obesity, metabolic syndrome, and type 2 diabetes mellitus pandemic: Part I: Increased cardiovascular disease risk and the importance of atherogenic dyslipidemia in persons with the metabolic syndrome and type 2 diabetes mellitus. J Cardiometab Syndr.

[3] Patra SK, Singh K, Singh R (2013). Paraoxonase 1: a better atherosclerotic risk predictor than HDL in type 2 diabetes mellitus. Diabetes Metab Syndr.

[4] Superko HR (2009). Cardiovascular event risk: high-density lipoprotein and paraoxonase. J Am Coll Cardiol.

[5] Sumner AE, Zhou J, Doumatey A, Imoisili OE, Amoah A, Acheampong J (2010). Low HDL-Cholesterol with Normal Triglyceride Levels is the Most Common Lipid Pattern in West Africans and African Americans with Metabolic Syndrome: Implications for Cardiovascular Disease Prevention. CVD Prev Control.

[6] Marsche G, Saemann MD, Heinemann A, Holzer M (2013). Inflammation alters HDL composition and function: implications for HDL-raising therapies. Pharmacol Ther.

[7] Gugliucci A, Menini T (2015). Paraoxonase 1 and HDL maturation. Clin Chim Acta.

[8] Davis KA, Crow JA, Chambers HW, Meek EC, Chambers JE (2009). Racial differences in paraoxonase-1 (PON1): a factor in the health of southerners?. Environ Health Perspect.

[9] Kalupahana NS, Moustaid-Moussa N, Claycombe KJ (2012). Immunity as a link between obesity and insulin resistance. Mol Aspects Med.

[10] Kanbak G, Akalin A, Dokumacioglu A, Ozcelik E, Bal C (2011). Cardiovascular risk assessment in patients with type 2 diabetes mellitus and metabolic syndrome: role of biomarkers. Diabetes Metab Syndr.

[11] Ogbera AO (2010). Prevalence and gender distribution of the metabolic syndrome. Diabetol Metab Syndr.

[12] Patel TP, Rawal K, Bagchi AK, Akolkar G, Bernardes N, da Silva Dias D (2016). Insulin resistance: additional risk factor in the pathogenesis of cardiovascular disease in type 2 diabetes. Heart Fail Rev.

[13] Pouliot MC, Depres JP, Nadeau A, Moorjani S, Prud´Homme D, Lupien PJ (1992). Visceral obesity in men. Associations with glucose tolerance, plasma insulin and lipoprotein levels. Diabetes.

[14] Lewis GF, Uffelman KD, Szeto LW, Weller B, Steiner G (1995). Interaction between free fatty acids and insulin in the acute control of very low density lipoprotein in Humans. J Clin Invest.

[15] Abate N, Garg A, Peshook RM, Stray-Gundersen J, Grundy SM (1995). Relationships of generalized and regional adiposity to insulin sensitivity in men. J Clin Invest.

[16] Hopkins GJ, Barter PJ (1986). Role of triglyceride-rich lipoproteins and hepatic lipase in determining the particle size and composition of high density lipoproteins. J Lipid Res.

[17] Mittendorfer B, Liem O, Patterson BW, Miles JM, Klein S (2003). What does the measurement of whole-body fatty acid rate of appearance in plasma by using a fatty acid tracer really mean?. Diabetes.

[18] Kirk EP, Klein S (2009). Pathogenesis and pathophysiology of the cardiometabolic syndrome. J Clin Hypertens (Greenwich).

[19] Liu ML, James RW, Ylitalo K, Taskinen MR (2004). Associations between HDL oxidation and paraoxonase-1 and paraoxonase-1 gene polymorphisms in families affected by familial combined hyperlipidemia. Nutr Metab Cardiovasc Dis.

[20] White R, Giordano S, Datta G (2017). Role of HDL-Associated Proteins and Lipids in the Regulation of Inflammation. Advances in lipoprotein research.

[21] Shunmoogam N, Naidoo P, Chilton R (2018). Paraoxonase (PON)-1: a brief overview on genetics, structure, polymorphisms and clinical relevance. Vasc Health Risk Manag.

[22] Ferré N, Feliu A, García-Heredia A, Marsillach J, París N, Zaragoza-Jordana M (2013). Impaired paraoxonase-1 status in obese children. Relationships with insulin resistance and metabolic syndrome. Clin Biochem.

[23] Goswami B, Tayal D, Gupta N, Mallika V (2009). Paraoxonase: a multifaceted biomolecule. Clin Chim Acta.

[24] Devarajan A, Shih D, Reddy ST (2014). Inflammation, infection, cancer and all that.the role of paraoxonases. Adv Exp Med Biol.

[25] Kalmár T, Seres I, Balogh Z, Káplár M, Winkler G, Paragh G (2005). Correlation between the activities of lipoprotein lipase and paraoxonase in type 2 diabetes mellitus. Diabetes Metab.

[26] Nair SP, Shah NC, Taggarshi A, Nayak U (2011). PON1 and its association with oxidative stress in type 1 and type 2 diabetes mellitus. Diabetes Metab Syndr.

[27] Mackness MI, Arrol S, Durrington PN (1991). Paraoxonase prevents accumulation of lipoperoxides in low-density lipoprotein. FEBS Lett.

[28] Durrington PN, Mackness B, Mackness MI (2001). Paraoxonase and atherosclerosis. Arterioscler Thromb Vasc Biol.

[29] Blatter Garin MC, Moren X, James RW (2006). Paraoxonase-1 and serum concentrations of HDL-cholesterol and apoA-I. J Lipid Res.

[30] Araoye MO (2004). Research Methodology with Statistics for Health Sciences, Nathadex Publishers. Ilorin.

[31] Uloko AE, Musa BM, Ramalan MA, Gezawa ID, Puepet FH, Uloko AT (2018). Prevalence and Risk Factors for Diabetes Mellitus in Nigeria: A Systematic Review and Meta-Analysis. Diabetes Ther.

[32] US Preventive Services Task Force; Davidson KW, Barry MJ, Mangione CM, Cabana M, Caughey AB (2021). Screening for Prediabetes and Type 2 Diabetes: US Preventive Services Task Force Recommendation Statement. JAMA.

[33] Adiga U, Banawalikar N, Menambath DT (2022). Association of paraoxonase 1 activity and insulin resistance models in type 2 diabetes mellitus: Cross-sectional study. J Chin Med Assoc.

[34] Suvarna R, Rao SS, Joshi C, Kedage V, Muttigi MS, Shetty JK (2011). Paraoxonase activity in type 2 diabetes mellitus patients with and without complications. J Clin Diagnostic Res.

[35] Patil Asmita B, Ganu Jayashree V (2013). Paraoxonase-1, total cholesterol and HDL cholesterol in diabetes mellitus. Indian J Basic Appl Med Res.

[36] Shah MM, Badade ZG (2016). Study of serum paraoxonase levels in type 2 diabetes mellitus patients & suggests risk of cardiovascular disease. Medical Science.

[37] Trentini A, Bellini T, Bonaccorsi G, Cavicchio C, Hanau S, Passaro A (2019). Sex difference: an important issue to consider in epidemiological and clinical studies dealing with serum paraoxonase-1. J Clin Biochem Nutr.

[38] Jeelani H, Tabassum N, Rashid F (2020). Association of Paraoxonase1 enzyme and its genetic single nucleotide polymorphisms with cardio-metabolic and neurodegenerative diseases. Gene Reports.

[39] Costa LG, Giordano G, Furlong CE (2011). Pharmacological and dietary modulators of paraoxonase 1 (PON1) activity and expression: the hunt goes on. Biochem Pharmacol.

